# C1-C2 instability in psoriatic arthritis

**DOI:** 10.11604/pamj.2020.36.217.24850

**Published:** 2020-07-27

**Authors:** Teka Maher, Zaier Akram Yassine, Ben Hnia Majdi, Naouar Nader

**Affiliations:** 1 Department of Orthopaedics and Trauma, Taher Sfar Hospital of Mahdia, Mahdia, Tunisia,; 2 Department of Orthopaedics and Trauma, Sahloul Hospital of Sousse, Sousse, Tunisia

**Keywords:** Arthritis psoriatic, spondyloarthropathies, arthrodesis

## Abstract

Cervical spine damage is common in psoriatic arthritis especially in older forms and it is rarely initiated by symptomatic atloid-axoid instability. Spinal involvement is frequently associated with sacroiliac dysfunction, the cervical spine involvement is observed in 35%-75% of cases with two types of radiological lesion. Upper cervical spine localization often manifests as C1-C2 arthritis, lower cervical spine involvement is manifested by syndesmophytes, ossification of the anterior longitudinal ligament and posterior inter apophyseal osteoarthritis. Our case is about a late onset upper cervical spine instability in a 45-year-old patient who has been treated for 20 years for rheumatism and has checked for paraesthesia's of the four limbs and gait difficulty that have been evolving over the last 3 months and the outcome of this case is that a C1-C2 instability must be systematically checked for in view of the appearance of deficient signs.

## Introduction

Cervical spine damage is common in psoriatic arthritis. It is most often seen in older forms of the disease and may or may not be associated with peripheral joint damage. Symptomatic atloid-axoid instability rarely initiates the disease. We report a case of late-onset upper cervical spine instability in psoriatic arthritis.

## Patient and observation

**Figure 1:**
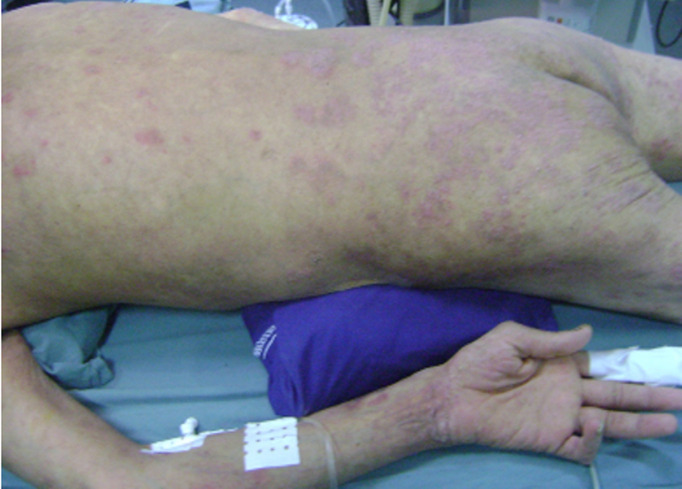
photo showing psoriatic skin lesions

**Figure 2:**
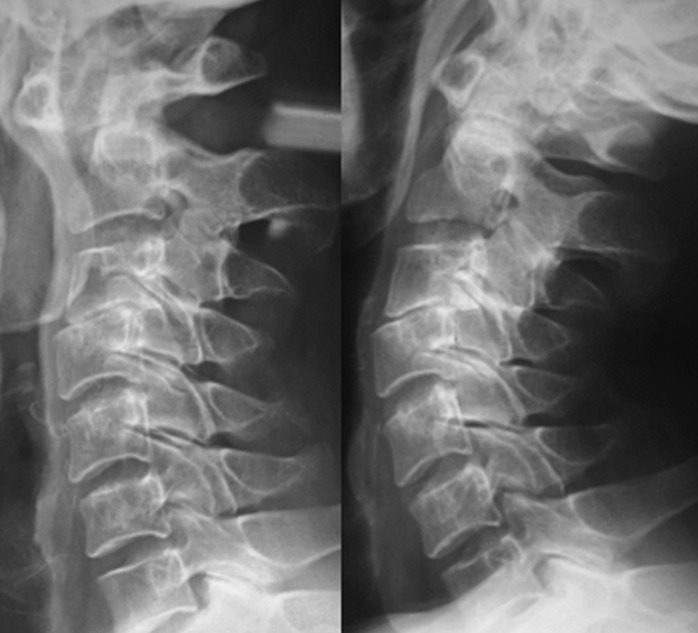
dynamic X-rays showing overt C1-C2 instability and C3/C4 retrolisthesis

**Figure 3:**
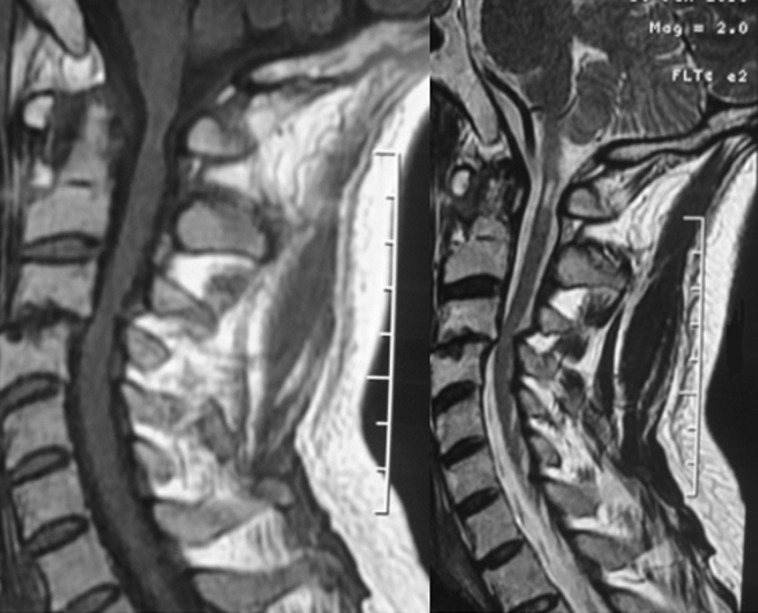
MRI appearance in T1 and T2 showing instability and signs of spinal cord pain

**Figure 4:**
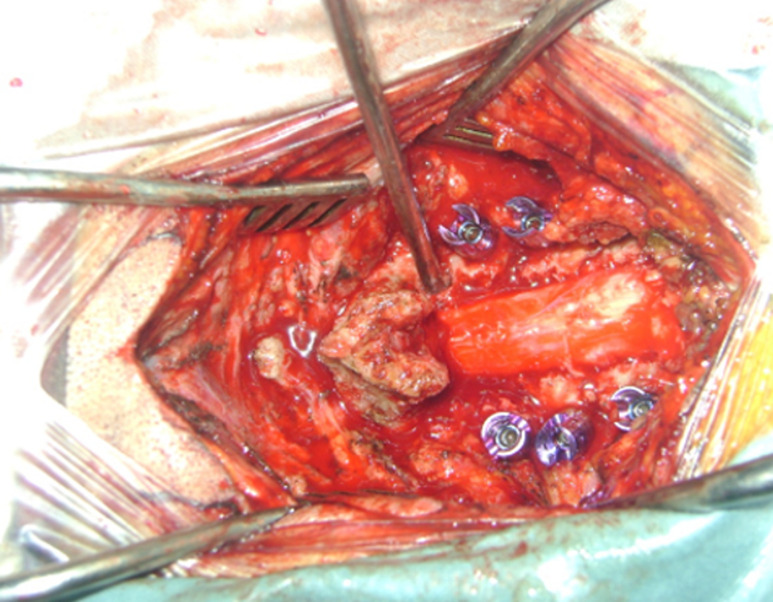
intraoperative view after laminectomy C3, C4, C5

**Figure 5:**
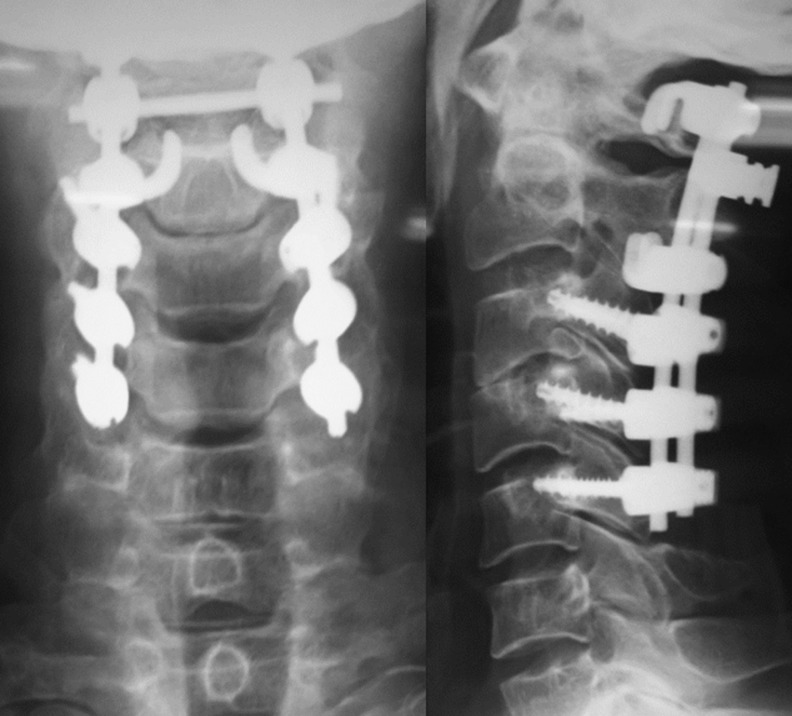
X-rays (6 months later)

A 45-year-old patient, treated for 20 years for psoriatic rheumatism using immunosuppressant drugs, who has checked for paraesthesia´s of the four-limb and gait difficulty that have been evolving over the last 3 months. Clinical examination revealed extensive skin lesions on the trunk and on all four limbs in the form of rounded or polycyclic red patches (Figure 1) and a pyramidal tract syndrome with predominantly left-sided muscle weakness. Standard X-rays of the cervical spine showed subtotal pinching of the C3-C4 intervertebral space with C3 posterior wall recoil. Dynamic X-rays showed obvious C1-C2 instability (Figure 2). Magnetic resonance imaging (MRI) showed damage to the transverse ligament resulting in C1-C2 dislocation (increased space between the odontoid and posterior arch of C1) and the presence of retrolisthesis of C3 relative to C4 with signs of spinal cord compression at this level (Figure 3). The patient had an instrumented posterior arthrodesis C1, C2, C3, C4 and C5 associated with laminectomy C3, C4 and C5 (Figure 4, Figure 5). At 6 months follow-up, the evolution was satisfactory with a complete neurological recovery and a good stability of the upper cervical spine.

## Discussion

Psoriatic arthritis belongs to the group of spondyloarthropathies. The prevalence of psoriasis in the general Caucasian population is 1-3% [1, 2]. It is rarer among Africans, Black Americans, Latin American, Indians and the Chinese population. The distribution is identical in men and women [2], with an average age of 27 years. The familial nature of psoriasis has been demonstrated by several studies [3]. Spinal involvement is frequently associated with sacroiliac dysfunction. It is characterized by the existence of syndesmophytes [4]. Cervical spine involvement in psoriatic arthritis is well known; it is observed in 35-75% of cases [5]. It occurs most often during severe psoriatic arthritis with a long evolutionary period [6, 7]. Two types of radiological lesions of the cervical spine are described in the literature: upper and lower cervical [8, 9]. Upper cervical spine localization, often manifests as C1-C2 arthritis with erosion of the odontoid in 10% of cases and as atloid-axoid dislocation in 5% of cases, which is classically of late occurrence and may be complicated by a fracture of the odontoid process or spinal cord compression. Lower cervical spine (LCS) involvement is manifested by syndesmophytes, ossification of the anterior longitudinal ligament and posterior inter apophyseal osteoarthritis [5]. It has been considered by many authors to be a non-life-threatening condition and rarely leads to a functional prognosis. This was suggested by the frequency of oligoarticular forms with often satisfactory evolution.

## Conclusion

Cervical spine involvement in psoriatic arthritis is frequent (35-75% of the cases) and shows a long evolution of the disease. A C1-C2 instability must be systematically checked for on the dynamic cervical spine X-ray, in view of the appearance of deficient signs.
